# What Accounts for Physical Activity during Pregnancy? A Study on the Sociodemographic Predictors of Self-Reported and Objectively Assessed Physical Activity during the 1st and 2nd Trimesters of Pregnancy

**DOI:** 10.3390/ijerph17072517

**Published:** 2020-04-07

**Authors:** Ana Mendinueta, Haritz Esnal, Haritz Arrieta, Miren Arrue, Nerea Urbieta, Itziar Ubillos, Kristina W. Whitworth, Xavier Delclòs-Alió, Guillem Vich, Jesus Ibarluzea

**Affiliations:** 1 Faculty of Medicine and Nursing, University of the Basque Country (UPV/EHU), 20014 Donostia-San Sebastián, Spain; HARITZ.ESNALAMUNDARAIN@osakidetza.eus; 2 Department of Nursing II, Faculty of Medicine and Nursing, University of the Basque Country (UPV/EHU), 20014 Donostia-San Sebastián, Spain; haritz.arrieta@ehu.eus; 3 Department of Obstetrics and Gynecology, Donostia University Hospital, Basque Country, 20014 Donostia-San Sebastián, Spain; MIREN.ARRUEGABILONDO@osakidetza.eus; 4 Biodonostia Health Research Institute, Group of Environmental Epidemiology and Child Development, Doctor Begiristain, s/n, 20014 San Sebastian, Spain; n-urbietamacazaga@euskadi.eus (N.U.); itziar.ubillos@gmail.com (I.U.); mambien3-san@euskadi.eus (J.I.); 5 Center for Precision Environmental Health, Department of Medicine, Baylor College of Medicine, Houston, TX 77030-3411, USA; Kristina.W.Whitworth@uth.tmc.edu; 6 Institute of Urban and Regional Development, University of California, Berkeley, CA 94720-1820, USA; Xavier.Delclos@uab.cat; 7 Department of Geography, UniversitatAutònoma de Barcelona, 08193 Cerdanyola del Vallès, Spain; Guillem.Vich@uab.cat; 8 Department of Health of the Basque Government, Subdirectorate of Public Health of Gipuzkoa, Avenida Navarra 4, 20013 Donostia-San Sebastian, Spain; 9 Spanish Consortium for Research on Epidemiology and Public Health (CIBERESP), Instituto de Salud Carlos III, C/Monforte de Lemos 3-5, 28029 Madrid, Spain; 10 Faculty of Psychology. University of the Basque Country (UPV/EHU), Avenida Tolosa 70, 20018 Donostia-San Sebastian, Spain

**Keywords:** accelerometer, self-reported data, physical activity guidelines, inter-measurement agreement, pregnancy

## Abstract

Physical activity (PA) during pregnancy has positive health implications for both mother and child. However, current literature indicates that not all pregnant women meet the international recommendations for PA (at least 150 min/week of moderate-to-vigorous PA). The main objective of this study was to assess PA levels among pregnant women in the city of Donostia-San Sebastian and identify their main sociodemographic predictors. We recruited 441 women in the 12th week of pregnancy from the local public obstetric health services. Women wore an accelerometer for one week during two separate time points (1st and 2nd trimesters of pregnancy) and completed a questionnaire assessing several sociodemographic variables as well as self-reported PA. With this information, we estimated women’s overall PA levels during both time points. The fulfillment of PA recommendations raised up to 77% and 85% during the first and second trimesters, respectively. We found that a higher number of children and a greater preference for exercise positively predicted light-to-moderate PA, being the most consistent predictors. The availability of a greater number of cars negatively predicted moderate-to-vigorous PA.

## 1. Introduction

### 1.1. Physical Activity during Pregnancy: On Guidelines, Performance, and Determinants

Regular physical activity (PA) is beneficial for health at all life stages [1,2,3]. Many studies, including several reviews and meta-analyses, have confirmed physical and emotional benefits during pregnancy [3,4,5,6,7,8], while the risks are scarce [9]. PA helps to avoid excessive weight gain during pregnancy, which additionally decreases the risks of comorbidities, such as gestational diabetes [3,10,11,12], macrosomia [7,13], preeclampsia [8,14], and assisted or caesarean delivery [15,16,17]. Moreover, PA helps to maintain—or increases—cardiovascular endurance, muscle strength, resistance, agility, coordination, and equilibrium [5,11,18] and reduces the impact of muscle-skeletal pain such as backache [19]. The emotional benefits of PA include the strengthening of one’s mood and self-esteem, increased sleep quality [3,20], and decreased stress during pregnancy [21].

PA recommendations for pregnant women have historically been focused on light PA intensities, under the assumption that vigorous activities might result in negative consequences for the mother and the child [22]. Current PA recommendations during pregnancy have been updated and are now similar to those for the general population [2], but modified to minimize maternal and fetal risks/injuries and maximize benefits [9,20]. Both the U.S. Department of Health and Human Services (US DHHS) and the World Health Organization (WHO) recommend pregnant women to perform 150 minutes per week of low impact aerobic exercise at moderate intensity [2,3,23]. National guidelines from countries like Canada, the United Kingdom (UK), Denmark, Norway, as well as those from the American College of Obstetricians and Gynecologists (ACOG), do not focus on overall time spent on PA in a given week but instead recommend 30 minutes of moderate exercise on 4–7 days per week [24]. The Spanish Society of Gynaecology and Obstetrics (SEGO) recommends moderate PA sessions in a frequency of 3–5 times per week with no duration specified. The WHO established that, in order to be considered as suitable, PA must occur in bouts of at least 10 minutes [2]. This consideration, set in the nineties [25], was included in most PA guidelines and reports. Nevertheless, a recent review of 25 studies [26] concluded that moderate PA contributes to health regardless of the episode’s duration, which has been reinforced in studies published thereafter [27,28] and included in current PA guidelines [3]. 

According to several studies, women tend to reduce their PA during pregnancy [29,30,31,32]. Available evidence also suggests that PA levels are lower in the 3rd trimester than in either the 1st or 2nd trimesters [24,29,30,33,34]. Despite its numerous benefits, studies report that only between a one-fifth [29,31,33,35,36] or one-third [30,37,38] of pregnant women meet PA recommendations. However, the former studies calculated those rates using time bouts. Within this literature, the study by Sytsma et al. [35] stands out due to the high proportion of US pregnant women (60%) classified as having met PA recommendations during the 1st trimester. However, it is not evident from the text of such paper whether or not the authors established any kind of time bout. 

The benefits ascribed to PA highlight the importance of identifying important (potentially modifiable) determinants of PA during pregnancy. In doing so, those at risk of inactivity could be identified and encouraged to develop a more active lifestyle during pregnancy. Studies which have thus far tried to identify determinants of PA among pregnant women have largely found positive associations between high educational attainment and household income and PA levels [8,31,35,39,40,41,42,43]. While many studies have also found that women with at least one previous child are more inactive during pregnancy [7,32,40,41,42,44,45], Watson et al. [46] found the opposite pattern of results; still others have found no statistically significant association between parity and PA during pregnancy [31,47]. The relationship between race and exercise is not consistent, with some studies finding that exercisers are more likely to be white [35,39,41], while others found similar exercise rates among black and white women [43,44]. Some others indicated that there is no relation between ethnic origin and PA [31,32,48]. Finally, job status, body mass index (BMI), and tobacco use did not show consistent associations with PA levels [45]. Nascimiento et al. [33] supported the results regarding educational level and parity exposed above and found a positive association between pre-pregnancy and pregnancy PA levels. The study also informed about the positive effect that receiving PA-related information has on the latter.

### 1.2. Measurement Options for PA, Advantages, and Disadvantages

The accurate measurement of PA is a critical issue. To date, questionnaires are the most common tool used to assess PA due to their ease of use and advantageous economic cost in comparison to other alternatives. However, the use questionnaires to quantify PA may result in misclassification because the PA evaluation often consists of the aggregation of questions addressing different activities (e.g., jogging or working out) [49]. Moreover, the frequency of reported activities may vary across time and participants often report the latest value (which may not be very indicative of their habitual performance) or just the opposite [49]. The wording of questions may also condition certain answers which might be also affected by external factors (e.g., question complexity, age, social desirability, etc.) [50]. 

Accelerometers are an interesting alternative to questionnaires because they provide an objective and more accurate measurement and they provide reliable and valid PA indices during pregnancy [51]. These electronic devices measure acceleration events (counts) and detect movements in real time by the use of the three axes (vertical, medio-lateral, and antero-posterior). Then, counts are transformed into the unit of interest for the researcher (e.g., Metabolic Equivalents of Task -METs-, minutes per day of a given PA intensity, or steps). These devices are usually placed on the hip or the waist. However, results depend on the applied cut-off points which can influence the measurement of PA intensity. An inherent limitation of some accelerometers is that they are not waterproof, and therefore, swimming and other water activities are not measurable [50,52]. 

Questionnaires and accelerometers are commonly used measurement tools of PA. There is some evidence showing low correlation between them. For instance, a systematic review by Skender et al. [53] reported correlations between questionnaire and accelerometer-based measurements of total PA ranging from r = 0.14 to r = 0.58 and observed that only one-third of studies had correlations over r = 0.40. More recently, a Canadian study showed a modest correlation (r = 0.36) between questionnaire and accelerometer-based PA [54]. Despite the former, another systematic review concluded that questionnaire measurements could lead to over- or underestimation (possibly due to cognitive biases) and reported a greater correlation range (from −0.71 to 0.96) [55]. Taking into account that each method measures different aspects and they provide complementary information, it is advisable to use them jointly in order to get complete information [53,56].

### 1.3. Study Aim

With this study, we aimed to: (1) describe the PA levels of pregnant women living in the study area (Metropolitan area of Donostia-San Sebastian, Basque Country, Spain) and quantify whether pregnant women in this area meet PA guidelines; (2) compare PA levels during the 1st and 2nd trimesters of pregnancy; (3) study the agreement between objective and reported PA measures; and, (4) determine which sociodemographic variables are predictive of PA levels during pregnancy. 

## 2. Materials and Methods 

### 2.1. Study Sample

For this population-based prospective study, we recruited 441 women (mean age 33.52, SD = 4.88) residing in the metropolitan area of Donostia-San Sebastián (Spain). This area, located in the Northeastern region of the Basque Country, is comprised of the municipalities of Astigarraga, Donostia-San Sebastián, Rentería, Hernani, Lasarte-Oria, Lezo, Oiartzun, Pasajes, Urnieta, and Usurbil. All these municipalities comprise the functional area of the main city of the region (Donostia-San Sebastián) and maintain a semi-continuous urban scene.

Participants were recruited among the women that attended the obstetric health service for the 12th week ultrasound that is routinely conducted in the Basque Public Health System. Exclusion criteria were: multiple pregnancy, not being able to adequately communicate in Basque, Spanish, or English, and not being resident in the study area during the whole study period. Women with high risk of a fetal chromosomopathy (>1/270) or those with an increased risk of a fetal cardiopathy due to increased nuchal fold in the first trimester (>p 95) were also excluded from the study. The follow-up of these women was done in a tertiary hospital different from our study center. If interested, eligible women were led to a private room where a researcher provided them with further information about the study and explained the implications of taking part in it. After signing the informed consent, participants were administered the study questionnaire and provided with one accelerometer (ActiGraph GT3X-BT; ActiGraph LLC, Pensacola, FL, USA). Participants were instructed to wear the accelerometer for 7 days starting on the day of recruitment and given information on how to return the device after its use. The accelerometer was worn over the hip and participants were asked to remove it when showering or engaging in water activities such as swimming and night-time sleeping. Prior to the 20th week of pregnancy and before attending the second ultrasound that is part of routine care, participants were re-contacted and invited to wear the accelerometer for an additional week. They were also given a short questionnaire. 

The study protocol was approved by the Research Ethics Committee of the Health Department of the Basque Government (Ref. no PI2018108).

### 2.2. Study Instruments and Variables

#### 2.2.1. Physical Activity

Objective physical activity was based on the use of a triaxial ActigraphwGT3X-BT set at 30 Hz and epoch length 60 seconds. Participants’ PA data was included in analyses if they had worn the device a minimum of three days with at least 10 hours of use per day. We defined non-wear as intervals of 90 minutes without recorded activity and sleeping hours (23:00-06:00), which were not taken into account. Freedson et al. (1998) [57] thresholds were used to calculate the minutes of light, moderate, or vigorous activity. For the present analyses, we built two composite measures reflecting light-to-moderate physical activity (LMPA) and moderate-to-vigorous physical activity (MVPA) including the total time spent in those PA categories.

A section of the questionnaire administered to women comprised six items extracted from the INMA (Infancia y Medio Ambiente—Childhood and Environment) cohort study’s battery test [58], which were used to assess PA. These items asked women to report the total weekly time devoted to a series of light (e.g., housework and yoga/pilates) and moderate (e.g., walking/biking, exercise/sport, swimming, and working out) PAs. Each of the questions was accompanied by five answer options comprising different time intervals, from < 1h/week to > 5h/week. Additionally, we included a question that asked participants to define themselves as sedentary, scarcely active, moderately active, quite active, or very active. 

#### 2.2.2. Covariates

The questionnaires (1st and 2nd trimester) allowed us to gather data on variables that may serve as determinants of PA among our study sample. We asked participants for their height and weight before and during pregnancy, which permitted us to calculate their Body Mass Index (BMI) in each of those time points and assign them to a specific BMI category (underweight, normal weight, overweight, and obese). The questionnaires also assessed whether the pregnancy was natural or assisted, as well as the mother’s nationality, educational attainment (primary, secondary, or university education), and current work situation (unemployed, homemaker, student, working, or on work leave). Finally, participants reported their age (years), the number of currently living children they had, the number of cars in the household, the number of hours they nap and watch TV during the week, tobacco and alcohol use during pregnancy (yes/no), the extent to which they like walking and exercising (1 = none to 5 = a lot), and the frequency they meet friends and relatives (1 = every day to 5 = never). 

### 2.3. Data Analysis

First, we described our sample’s PA levels and assessed whether our participants had fulfilled the PA guidelines set forth by the US DHHS and the WHO [2,3]. We also assessed the agreement between self-reported (i.e., questionnaire-based) and objective (i.e., accelerometer-based) measures of PA. Second, we used Spearman’s correlation and simple linear regression models to examine the relationship between self-reported and objective measures of PA, for which we also applied ordinary least squares and model validation via inspection of residuals. In order to analyze the degree of agreement between both measures, we used the package psy [59] in the R software v. 3.6.1 [54] to estimate Cohen’s Kappa (Cohen 1960) and the intraclass correlation coefficient (ICC) [60], both with bootstrap-based 95% confidence intervals [61]. Third, we used repeated measures t-tests to evaluate 1st and 2nd trimester PA levels and detect possible statistically significant differences. Finally, we aimed at presenting summaries of bivariate analysis, for which we used Spearman’s correlations to examine the association between continuous self-reported/objective continuous measures of physical activity and a set of potentially explanatory continuous variables (e.g., x, y, z). Besides, we used F-tests, together with Dunnet post-hoc comparisons, to examine the association between the same measures of physical activity and potentially explanatory categorical variables (e.g., x, y, z). We did so to build predictive models of PA using the set of sociodemographic and background variables. Thus, we applied stepwise-based model selection, parameter estimation through ordinary least squares, and model validation via inspection of residuals, to build predictive linear models for the self-reported/objective continuous measures of physical activity. These standard statistical techniques [62,63] were applied using R software v. 3.6.1 [64].

## 3. Results

**Figure 1:**
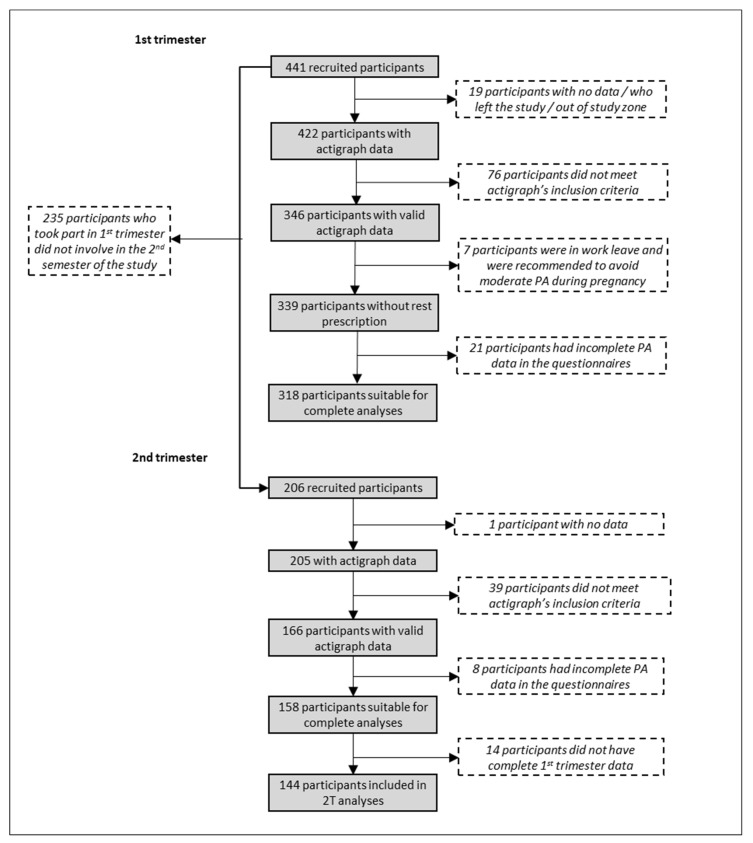
Flowchart showing participants included in the 1st and 2nd trimester analyses.

We recruited 441 women during the 1st trimester of pregnancy, of which 206 took part in the 2nd trimester’s measurement as well. Mostly due to uncomplete data or scarce Actigraph use, around 25% of participants by timepoint were removed from analyses. Figure 1 depicts the number of participants available for the analyses and the causes of participants’ exclusion. As explained in the previous section, we had available data from 339 participants for the analyses. Participants were 33.78 years old on average (SD = 4.40). Half of the sample (50.44%) had no currently living children previous to the ongoing pregnancy, 36.87% had one previous child, and the rest had two or more other children. Two-thirds of women (66.67%) were classified as having normal weight, 16.22% were overweight, and 6.78% were obese. Most of the study sample had completed tertiary education (64.60%) and were employed and working at the time of the study (79.94%). The sociodemographic profile of the study participants can be found in Supplementary Table S1.

### 3.1. PA Levels by Trimester and Fulfillment of International Recommendations

According to our measures, participants spent much more time in activities considered light to moderate than moderate to vigorous. This was true for both the objective and self-reported measures as well as for the two trimesters of pregnancy. Table 1 presents participants’ LMPA and MVPA levels during the first trimester of pregnancy as determined by the questionnaire and the accelerometer.

We also used both self-reported estimates and objective measures of moderate physical activity during the first and second trimesters of pregnancy to assess the extent to which the standard recommendations of practicing moderate exercise during at least 150 minutes per week were fulfilled by the participants in the sample. When considering self-reported estimates, 244 out of 315 (77.5%; first trimester) and 134 out of 158 (84.8%; second trimester) declared to fulfil this standard recommendation, respectively. On the other hand, when considering objective measures, 264 out of 339 (77.9%) and 141 out of 166 (84.9%) fulfilled the recommendation during the first and second trimester, respectively. Hence, both methods identified a comparable number of participants meeting the recommendations.

### 3.2. Comparison of PA Levels between the First and Second Trimesters of Pregnancy

To assess whether participants’ PA decreased between the 1st and 2nd trimesters, we compared the four outcome measures for the subsample of participants who had complete data at both time points. This analysis, shown in Table 2, revealed that PA levels remained the same in both trimesters except for SLMPA, which was higher in the 2nd trimester. The size of this difference was small (*d* = 0.37). 

### 3.3. Associations and Agreement between Objective and Self-Reported Measures

**Figure 2:**
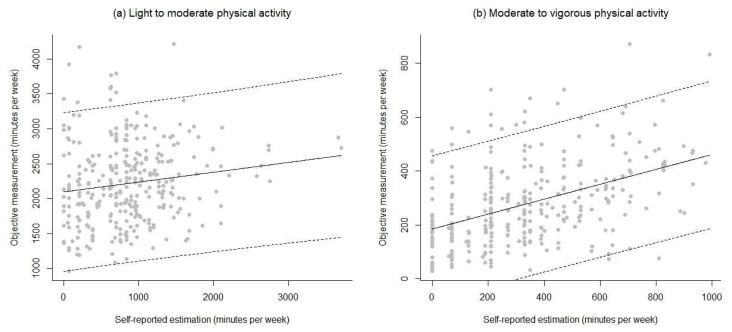
Regression models’ residuals for LMPA and MVPA (self-reported and objective) for the first trimester of pregnancy.

**Figure 3:**
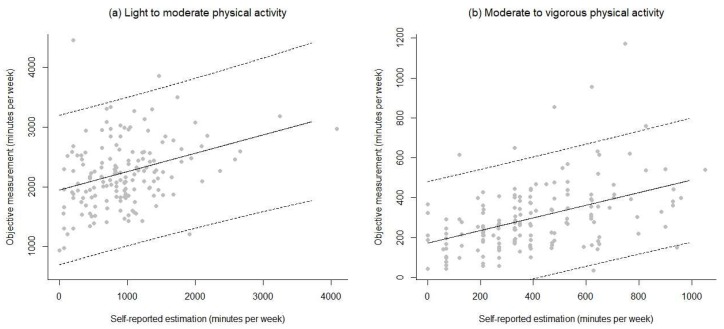
Regression models’ residuals for LMPA and MVPA (self-reported and objective) for the second trimester of pregnancy.

Correlations between objective and self-reported scores were small in size for LMPA in each trimester (r = 0.15 and 0.31, respectively) and moderate for MVPA (r = 0.44 and 0.46, respectively). Based on this analysis, it appears that the subjective estimation LMPA should not be used to predict the objective estimation, since the fitted relationship between both variables explains just adj.-R^2^ = 1.8% (Figure 2a). The subjective estimation of MVPA is a poor predictor of the objective measurement, because the fitted relationship explains only R^2^ = 18.9% (Figure 2b). In the case of the 2nd trimester, analyses lead to similar conclusions. The subjective estimation of LMPA cannot be used to predict the objective measurement of the same quantity, because it explains just adj.-R^2^ = 7.9% (Figure 3a); likewise, the subjective estimation of MVPA is a poor predictor (R^2^ = 19.1%) of the objective measurement (Figure 3b). 

Regarding the agreement between self-reported estimates and objective measures during the first trimester of pregnancy, 203 out of 318 were congruently classified by both measurement procedures—Cohen’s Kappa for agreement = 0.242 with bootstrap-based 95% C.I. (0.111, 0.361) and intraclass correlation coefficient (agreement version) = 0.242 with 95% C.I. (0.125, 0.354). In the second trimester, 120 of the 144 got classified as meeting the recommendations. This time, Cohen’s Kappa for agreement was 0.312 with bootstrap-based 95% C.I. (0.135, 0.521), and the intraclass correlation coefficient (agreement version) was 0.314 with 95% C.I. (0.118, 0.538). 

### 3.4. Predictive Models of PA during Pregnancy

Prior to specifying regression models, we conducted correlation analyses and F-tests to explore the association between the sociodemographic variables and the PA levels among our participants. Supplementary Table S2 shows the Spearman’s correlation estimates between continuous sociodemographic variables and 1st trimester PA variables. Supplementary Tables S3 and S4 present the distribution of the categorical sociodemographic variables and their associations with 1st trimester PA variables. Supplementary Tables S5 and S6 depict the same information regarding the second trimester of pregnancy. 

The predictive models for SLMPA, SMVPA, OLMPA, and OMVPA in the 1st and 2nd trimesters of pregnancy explained around one-fifth of the outcome variable’s variance (R^2^ = 0.15−0.21). The number of currently living children (previous to current pregnancy) was positively associated to LMPA (both objective and self-reported), whereas a greater number of cars in the household was negatively related to SLMPA, SMVPA, and OMVPA. Participants in situations other than working showed greater levels of SLMPA. In the case of OMVPA, only the participants who were employed but on maternal leave were more active than their employed and working counterparts. TV hours were also negatively associated to OLMPA. Finally, preference for walking and for exercise were positively associated to OMVPA and SLMPA (only exercise). 

The predictive models for the second trimester had an R^2^ between a 0.10 and 0.29, with the models on self-reported variables showing a better performance (in terms of explained variance) than the objective ones. Again, the number of children was positively associated with SLMPA but negatively with OMLPA. Domestic work was positively associated with SLMPA (compared to participants who were working) and participants employed but on maternal leave had more SMVPA than participants who were working. TV hours were positively associated with SLMPA. Preference for walking positively predicted OMVPA and preference for exercise did so for SLMPA and SMVPA. Curiously, car ownership did not play any role, and civil status appeared as a positive predictor of OMVPA, with single participants showing greater rates than married ones. All the regression models, including the variable coefficients and percentage of explained variance are shown in Table 3.

## 4. Discussion

### 4.1. PA Levels during Pregnancy and Agreement Between Self-Reported and Accelerometer Data

We aimed to assess whether PA levels among pregnant women in the metropolitan area of Donostia-San Sebastián meet international guidelines and to identify predictors of objective and self-reported estimations of PA among this population [2,3]. On average, objectively assessed MVPA during the first trimester was 278 min/week, while the self-reported amount was slightly higher (348 min/week). Time devoted to PA was similar during the second trimester. However, in the case of LMPA, objective estimations were remarkably greater than self-reports. Obviously, devices gather information about daily activities (e.g., climbing the stairs, taking rubbish out, etc.) which are not frequently included in questionnaires and that are therefore difficult to quantify with self-reported strategies. The proportion of women reporting PA levels that meet international guidelines in our study are remarkably greater than has been reported in much of the literature [29,30,31,35,37,38], with the exception of the study by Sytsma and colleagues [39]. However, it should be acknowledged that studies reporting relatively lower PA typically remove periods of time below 10 minutes from their PA calculation. This habit, which has been recently criticized [7,8,9], might have reduced the total sum of MVPA reported in previous studies. For our study, the use of time bouts reduces the fulfilment rate to 30% in the first trimester and 32.5% in the second, a picture closer to the ones found by previous researchers in other places of the world and reported above.

Another goal of the present study was to contribute to the ongoing comparisons and discussions about the use of objective and or self-reported measures of PA. Our data, showing small-to-moderate correlation between objective and self-reported measures of PA is consistent with a review indicating similar correlations ranging from 0.14 to 0.58 [53]. All the same, correlation indices only show the strength of the association between variables and should be complemented with some measure of agreement. In our study, Kappa statistics and ICCs revealed a low level of agreement between objective and self-reported measures of PA. These results are not unsurprising given that, as explained above, objective measurements register all movements and activities carried out by the individual and self-reported measures only the activities asked about. Hence, the use of each methodology (or both) should depend on the nature and objectives of the study to be conducted.

### 4.2. Determinants of PA during the 1st and 2nd Trimesters of Pregnancy

We also constructed predictive models to understand to what extent sociodemographic variables might account for observed PA levels and to identify potential fostering elements and barriers for exercise during pregnancy and further identify groups of women at risk of inactivity. In general terms, we found that the number of children, car ownership, work situation, and preference for exercise and walking were the most consistent predictors. Contributing less to the overall variance explained were TV hours, educational attainment, and civil status. 

The number of previous children was positively associated with LMPA in both trimesters and exerted a negative predictive role of MVPA during the second trimester. Despite the fact that this finding contradicts previous studies [7,32,40,41,42,44,45], Gaston and Cramp [45] stated that women with a higher number of children may have less time to devote to exercising and at the same time, maternity-related daily activities entail considerable amounts of PA and energy expenditure, which may explain our finding. Car ownership was also negatively associated with LMPA and MVPA levels during the first trimester, though no association was observed with PA in the second trimester. This may be related to daily trips that are completed by car instead of by, for example, walking or biking. Our finding that work situation was an important predictor of PA is in line with the study by Pereira et al., who found that longer work hours limit the ability to exercise [32]. Downs and colleagues [30] recognized that the experience of pleasure could be a relevant trigger for PA during pregnancy. We found support to this assertion as, in our study, preference for walking positively predicted OMVPA in both trimesters and preference for exercise predicted SLMPA (both trimesters) and SMVPA (second trimester). In line with some previous studies [40,43], ours revealed that lower educational level was associated to greater OLMPA, although this finding is not consistent in the literature [28,32,36,38]. Low-income citizens in South-European cities seem to engage in daily walking more often than affluent citizens [65], perhaps given the greater difficulty in accessing private transportation. Besides, their concentration in low-qualified and physically demanding jobs may increase their PA levels and then help to explain our results. 

### 4.3. Strengths, Limitations, and Future Lines of Research

The strength of this study is in: (1) the use of both objective and self-reported data to determine PA levels [53,56]; (2) the device-based determination of PA as triaxialaccelerometry is the most used, reliable, and valid option [50]; (3) the collection of data from the first and second trimesters of pregnancy; and (4) the prospective study design. 

Some limitations have to be acknowledged as well. First, self-reported data may result in misclassification of PA, particularly when separate questions addressing different domains or activities are used [49]. Similarly, we calculated BMI scores using self-reported height and weight data, which might be less reliable than objective measurement. Second, the PA questions we used are different from ones used in other studies and thus comparison is difficult. Third, a minimum of 4 days of valid use (at least 10 hours per day) has been frequently used as inclusion criteria for PA measurements [52], but we applied an at-least-3-days rule, that has been considered in different studies [46,66], although less frequently. Finally, the results of the study might be affected by a selection bias due to the fact that participants, recruited from all the pregnant women attending the obstetric public service of Donostia-San Sebastián, participated voluntarily and may not be fully representative of that population. This selection bias may have an effect also in participants’ attrition rate from 1st to 2nd trimesters (around 50%) because it might be expected that 2nd trimesters participants were among the most motivated and most interested in PA and health issues among our study sample. This may have probably led to an overestimation of the actual fulfillment of the PA recommendations for the second trimester.

## 5. Conclusions

We recruited 441 pregnant women to analyze PA patterns during the first and second trimesters of pregnancy and found that most of the women in our sample (around 80%) met the PA guidelines (at least 150 min/week of moderate-to-vigorous PA). We identified that self-reported and accelerometer-based PA measurements were not well correlated, which is consistent with previous literature and adds to the established recommendation of using complementary methods to wholly understand and analyze PA. In our study, the most important predictors of PA were number of children, car ownership, work situation, and preference for walking and exercising. However, in order to amplify the predictive power of models, and more importantly, the understanding of this question, future studies should incorporate variables at the psychological, social, and urban design spheres, including larger cities and semi-urban and rural areas representing different cultural backgrounds. 

## Figures and Tables

**Table 1:** 1st and 2nd trimesters’ physical activity (PA) levels (min/week) according to both subjective (self-reported) and objective (accelerometer-based) methods.

Variable	N	N*	Mean	SE Mean	SD	Min.	Max.	Q1	Median	Q3
**1st Trimester**										
SLMPA	314	25	875.9	14.2	252.3	0	1110	190	330	525
OLMPA	339	0	2239.5	34.1	604.1	0	3710	390	840	1200
SMVPA	315	24	348.7	8.2	151.6	28.	871.5	168	253.7	374.5
OMVPA	339	0	278.7	31.9	588	962.5	4214	1804.6	2202	2622
**2nd Trimester**										
SLMPA	158	195	975.5	49.8	626.2	0	4080	528.8	900	1287.5
OLMPA	166	187	2242.9	50.4	649.3	938	6501.3	1830.4	2167.8	2575.3
SMVPA	158	195	425.9	20.5	258	0	1110	210	390	620
OMVPA	166	187	295.2	13.1	169.1	35	1172	180.5	266	384.4

Note: Mean, standard error, standard deviation, minimum and maximum, Q1, median, and Q3 scores for study variables. N = Number of cases, N* = missing cases, SLMPA = Self-reported Light-to-Moderate Physical Activity, SMVPA = Self-reported Moderate-to-Vigorous Physical Activity, OLMPA = Objective Light-to-Moderate Physical Activity, OMVPA = Objective Moderate-to-Vigorous Physical Activity.

**Table 2:** Comparison of self-reported and accelerometer-based reports of PA levels, by trimester of pregnancy.

Pair of Variables	N	Mean	SD	SE	Mean Difference	SD*	SE*	95% C.I.	*t*-Value	*p*-Value
1TSLMPA	140	871.8	566	47.8	−97.6	526.3	44.5	(−185.6, −9.7)	−2.20	0.030
2TSLMPA	140	969.5	629.1	53.2						
1TOLMPA	151	2226.2	558	45.4	6.9	135.1	11	(−14.8, 28.6)	0.63	0.530
2TOLMPA	151	2238	555.5	45.2						
1TSMVPA	140	393.8	261.7	22.1	−31.6	204.2	17.3	(−65.8, 2.5)	−1.83	0.069
2TSMVPA	140	425.4	263.1	22.2						
1TOMVPA	151	304.9	155.9	12.7	−11.8	393.9	32.1	(−75.2, 51.5)	−0.37	0.713
2TOMVPA	151	298	164.7	13.4						

Note: Variables preceded by 1T and 2T correspond to the first and second trimester, respectively. SLMPA = Self-reported Light-to-Moderate Physical Activity, SMVPA = Self-reported Moderate-to-Vigorous Physical Activity, OLMPA = Objective Light-to-Moderate Physical Activity, OMVPA = Objective Moderate-to-Vigorous Physical Activity. SD* = Standard Deviation of the mean difference, SE* = Standard Error of the mean difference.

**Table 3:** Predictive models of SLMPA, SMVPA, OLMPA and OMVPA for the first and second trimesters of pregnancy.

1st Trimester of Pregnancy					2nd Trimester of Pregnancy				
	Coef.	95% C.I. Coef.	*p*−Value	Contribution to R ^2^		Coef.	95% C.I. Coef.	*p*−vValue	Contribution to R ^2^
**Response variable SLMPA**					**Response variable SLMPA**				
Constant	462	(137, 786)	0.005		Constant	308	(−37, 653)	0.080	
Number of children	209.70	(120.5, 298.80)	<0.001	8.69%	TV Habit	17,72	(5.75, 29.69)	0.004	1.72%
Preference for exercise	101.20	(42.9, 159.40)	0.001	3.12%	Preference for exercise	142,20	(43.50, 240.90)	0.005	8.34%
Car ownership	−105.50	(−207.7, −3.30)	0.043	2.44%					
Work Situation: Employed and Active	REF			5.64%	Work Situation: Employed and Active	REF			19.20%
Unemployed	229	(−23, 482)	0.075		Unemployed	−300	(−799, 199)	0.236	
Student	796	(26, 1566)	0.043		-				
Domestic work only	634	(318, 950)	<0.001		Domestic work only	1605	(1052, 2157)	<0.001	
Employed on maternal leave	107	(−180, 395)	0.463		Employed on maternal leave	119	(−298, 536)	0.573	
**Total explained variance**				**19.89%**	**Total explained variance**				**29.26%**
**Response variable OLMPA**					**Response variable OLMPA**				
Constant	2524	(2097, 2951)	<0.001		Constant	1788	(1587, 1989)	<0.001	
Number of children	252.40	(166.6, 338.10)	<0.001	13.84%	Number of children	295.10	(175.30, 414.80)	<0.001	13.81%
TV habit	−9.70	(−16.18, −3.22)	0.003	1.55%					
Education level	−170	(−267.8, −72.20)	0.001	3.08%					
**Total explained variance**				**18.47%**	**Total explained variance**				13.81%
**Response variable SMVPA**					**Response variable SMVPA**				
Constant	147.10	(28.80, 265.40)	0.015		Constant	216.70	(58.90, 374.40)	0.007	
Preference for exercise	107.70	(83.90, 131.40)	<0.001	19.88%	Preference for exercise	103.50	(61.90, 145.20)	<0.001	15.17%
Car ownership	−44.00	(−84.30, −3.70)	0.032	1.18%					
					Number of children	−84.40	(−143.90, −24.80)	0.006	2.54%
					Work Situation				4.60%
					Employed and Active	REF			
					Unemployed	57.40	(−125.70, 240.50)	0.536	
					Domestic work only	168	(−78, 413)	0.179	
					Employed on maternal leave	220.60	(46.80, 394.50)	0.013	
**Total explained variance**				**21.06%**	**Total explained variance**				**22.31%**
**Response variable OMVPA**					**Response variable OMVPA**				
Constant	149.70	(57.8, 241.60)	0.001		Constant	99.70	(−23.20, 222.60)	0.111	
Preference for walking	57.79	(40.12, 75.47)	<0.001	11.72%	Preference for walking	48.10	(15.40, 80.80)	0.004	4.64%
Car ownership	−29.10	(−55.1, −3.20)	0.028	1.06%	Civil status				5.64%
Work Situation: Employed and Active	REF			2.61%	Married (REF)	REF			
Unemployed	−1.20	(−65.1, 62.80)	0.971		Living with a partner	57	(−4.6, 118.5)	0.069	
Student	44.30	(−118.6, 207.20)	0.593		Divorced or separated	−17	(−337, 303)	0.916	
Domestic work only	−55.40	(−131.4, 20.50)	0.152		Single	118.5	(29.5, 207.4)	0.009	
Employed on maternal leave	95	(22.80, 167.30)	0.01						
**Total explained variance**				**15.39%**	**Total explained variance**				**10.28%**

Note: VIF = Variance Inflation Factor. SLMPA = Self-reported Light-to-Moderate Physical Activity, SMVPA = Self-reported Moderate-to-Vigorous Physical Activity, OLMPA = Objective Light-to-Moderate Physical Activity, OMVPA = Objective Moderate-to-Vigorous Physical Activity.

## Supplementary Materials

The following are available online at https://www.mdpi.com/1660-4601/17/7/2517/s1, Supplementary Table S1. Distribution of 1st trimester categorical and ordinal sociodemographic variables. Supplementary Table S2. Spearman correlations between 1st trimester PA levels and continuous sociodemographic variables. Supplementary Table S3. PA levels during 1st trimester of pregnancy by levels of categorical and ordinal sociodemographic variables. Supplementary Table S4. 2nd trimester PA levels and continuous sociodemographic variables. Supplementary Table S5. Spearman correlations between 2nd trimester PA levels and continuous sociodemographic variables. Supplementary Table S6. PA levels during 2nd trimester of pregnancy by levels of categorical and ordinal sociodemographic variables.
