# Supplementary material for: What Accounts for Physical Activity during Pregnancy? A Study on the Sociodemographic Predictors of Self-Reported and Objectively Assessed Physical Activity during the 1st and 2nd Trimesters of Pregnancy
Source: Int J Environ Res Public Health. 2020 Apr 7;17(7):2517. doi: 10.3390/ijerph17072517 (PMC7177875; doi:10.3390/ijerph17072517)
Supplement: Supplementary file 1 [file ijerph-17-02517-s001.pdf]

Supplementary Table 1. Distribution of 1st trimester categorical and ordinal sociodemographic variables.

Variable	N	Percentage
**Country of origin**		
Spain	277	81.7
America	39	11.5
Other	8	2.4
Unspecified	15	4.4
**Number of children**		
One	171	50.4
Two	125	36.9
Three	26	7.7
Four or more	5	1.5
Unspecified	12	3.5
**Body Mass index**		
Underweight	10	2.9
Normal	226	66.7
Overweight	55	16.2
Obese	23	6.8
Unspecified	25	7.4
**Natural pregnancy**		
No	36	10.6
Yes	281	82.9
Unspecified	22	6.5
**Civil status**		
Married	189	55.8
Living with a partner	88	26.0
Divorced or separated	4	1.2
Single	40	11.8
Respondent does not answer	4	1.2
Unspecified	14	4.1
**Education level**		
No education	2	0.6
Primary education	19	5.6
Secondary education	86	25.4
Tertiary education	219	64.6
Unspecified	13	3.8
**Working situation**		
Employed and active	271	79.9
Unemployed	18	5.3
Student	3	0.9
Domestic work only	15	4.4
Retired	16	4.7

Supplementary Table 1 (cont.). Distribution of 1st trimester categorical and ordinal sociodemographic variables.

Variable	N	Percentage
Employed on sick leave	5	1.5
Unspecified	11	3.2
**Car ownership**		
No car	25	7.4
One car	149	44.0
Two or more cars	155	45.7
Unspecified	10	2.9
**Tobacco smoking**		
Never Smoker	183	54.0
Current smoker	19	5.6
Non-daily smoker	7	2.1
Ex-smoker	120	35.4
Unspecified	10	2.9
**Alcohol drinking**		
Abstemious	120	35.4
Occasional drinker	181	53.4
Weekly drinker	27	8
Daily drinker	1	0.3
Unspecified	10	2.9
**Preference for walking**		
Never	4	1.2
Rarely	46	13.6
Sometimes	124	36.6
Often	109	32.2
Always	32	9.4
Unspecified	24	7.1
**Preference for exercise**		
Never	32	9.4
Rarely	85	25.1
Sometimes	133	39.2
Often	49	14.5
Always	28	8.3
Unspecified	12	3.5
**Contact with family/friends**		
Daily contact	177	52.2
Usual contact	129	38.1
Occasional contact	17	5
Unfrequent contact	4	1.2
Respondent does not answer	2	0.6
Unspecified	10	2.9

Supplementary Table 2. Spearman correlations between 1st trimester PA levels and continuous sociodemographic variables.

	SMVPA	SLMPA	OMVPA	OLMPA
SLMPA	**.59*** **			
OMVPA	**.44*** **	**.23*** **		
OLMPA	-.02	**.15** **	**.27*** **	
Age	.05	.10	<.01	.04
Weight prior to pregnancy	**-.15** **	**-.13***	-.09	-.05
Weight in the first trimester	**-.12***	-.10	-.06	-.03
Height	.01	-.03	.03	**-.12***
Sleep	.03	.03	< -.01	-.02
Siesta	.01	.05	-.04	-.05
TV habit	**.14***	**.11***	-.08	**-.20*** **

Note: Statistically significant values are highlighted in bold. * = $p < .05$, ** = $p < .01$ and *** = $p < .001$. *SLMPA* = Self-reported Light to Moderate Physical Activity, *SMVPA* = Self-reported Moderate to Vigorous Physical Activity, *OLMPA* = Objective Light to Moderate Physical Activity, *OMVPA* = Objective Moderate to Vigorous Physical Activity.

Supplementary Table 3. PA levels during 1st trimester of pregnancy by levels of categorical and ordinal sociodemographic variables.

	SLMPA				OLMPA				SMVPA				OMVPA			
**Contact with family/friends**	N	N*	Mean	SD	N	N*	Mean	SD	N	N*	Mean	SD	N	N*	Mean	SD
Daily contact (REF)	167	10	905.7	657.5	177	0	2185.6	561.2	168	9	349.6	236.3	177	0	278.7	160.5
Usual contact	126	3	838.7	529.4	129	0	2272.9	583	126	3	352.3	274.2	129	0	283.2	143.3
Occasional contact	16	1	740.6	293.9	17	0	2304	578	16	1	324.4	216.4	17	0	244.6	142
Unfrequent contact	3	1	**1990***	596	4	0	2227	477	3	1	420	471	4	0	300.1	157.6
Respondent does not answer	2	0	140	99	2	0	2717	2058	2	0	140	99	2	0	305	236
Unspecified	0	10	*	*	10	0	2563	776	0	10	*	*	10	0	262.1	120.7
**Preference for exercise**																
Never (REF)	31	1	675.5	401.3	32	0	2210	696	31	1	165.5	127.7	32	0	213.1	139.3
Rarely	80	5	739	577.6	85	0	2284.2	637.3	80	5	242.9	197.5	85	0	244.9	140.8
Sometimes	128	5	931.3	637.9	133	0	2210.8	550.8	128	5	**372.1***	250.2	133	0	277.1	148
Often	47	2	981.7	620.2	49	0	2212.1	518.3	48	1	**463.1***	218.2	49	0	**355.4***	169.2
Always	26	2	**1075***	599	28	0	2217.2	522.9	26	2	**577.9**	295.9	28	0	**336.5***	134.8
Unspecified	2	10	830	14.1	12	0	2484	760	2	10	200	14.1	12	0	262	110.1
**Preference for walking**																
Never (REF)	4	0	775	168.2	4	0	2006	539	4	0	145	168.2	4	0	185.8	87.9
Rarely	44	2	701.1	574.5	46	0	2194	744	44	2	195.9	181	46	0	172.2	103.5
Sometimes	119	5	847.1	629.8	124	0	2212.1	548.2	120	4	285.8	226.4	124	0	259.5	132.9
Often	105	4	976.6	576.8	109	0	2270.3	553.4	105	4	**449.4***	245.1	109	0	329.6	170.3
Always	29	3	925	717	32	0	2161.2	530.1	29	3	**510.2***	285.8	32	0	333.1	136.4
Unspecified	13	11	839.2	355.8	24	0	2472	671	13	11	336.2	175.9	24	0	293	132.3

Supplementary Table 3 (cont.). PA levels during 1st trimester of pregnancy by levels of categorical and ordinal sociodemographic variables.

	SLMPA				OLMPA				SMVPA				OMVPA			
**Alcohol drinking**	N	N*	Mean	SD	N	N*	Mean	SD	N	N*	Mean	SD	N	N*	Mean	SD
Abstemious (REF)	113	7	914.5	609	120	0	2274.1	538.7	114	6	367.8	258.5	120	0	294	150.5
Occasional drinker	174	7	885.3	622.8	181	0	2224.2	600.5	174	7	342	255.1	181	0	267.4	158.9
Weekly drinker	26	1	646.5	401.7	27	0	2066	618	26	1	315.4	208.8	27	0	287.2	112.9
Daily drinker	1	0	840	*	1	0	2301.3	*	1	0	210	*	1	0	409.5	*
Unspecified	0	10	*	*	10	0	2563	776	0	10	*	*	10	0	262.1	120.7
**Tobacco smoking**																
Never Smoker (REF)	175	8	899.4	635.2	183	0	2199.9	582.4	176	7	349.7	261.2	183	0	283.4	144.5
Current smoker	18	1	915	453	19	0	2438	631	18	1	371.4	253.7	19	0	247.6	129.7
Non-daily smoker	7	0	1302	411	7	0	2325	624	7	0	432.1	203.5	7	0	321.8	175.5
Ex-smoker	114	6	807.5	576.9	120	0	2236.5	565.6	114	6	338.5	242.4	120	0	275.3	166.9
Unspecified	0	10	*	*	10	0	2563	776	0	10	*	*	10	0	262.1	120.7
**Car ownership**																
No car (REF)	22	3	993	601	25	0	2230	521	23	2	370.2	217.4	25	0	316.9	129.1
One car	142	7	964	693	149	0	2244.9	610	142	7	371	263.7	149	0	295.9	158.5
Two or more cars	150	5	775.4	491.6	155	0	2215	562.5	150	5	324.4	245.5	155	0	256.9	147.8
Unspecified	0	10	*	*	10	0	2563	776	0	10	*	*	10	0	262.1	120.7
**Education level**																
No education (REF)	2	0	1283	456	2	0	2523	740	2	0	623	499	2	0	458.8	40.1
Primary education	17	2	962	634	19	0	2546	801	17	2	229.7	270.5	19	0	240.6	130
Secondary education	80	6	898.8	566.4	86	0	2373.1	637.8	81	5	356.6	269	86	0	258.9	156.3
Tertiary education	212	7	864.6	616.9	219	0	2133.3	499.7	212	7	356.3	240.1	219	0	289.6	152.9
Unspecified	3	10	303	466	13	0	2653	795	3	10	93.3	106.9	13	0	252.7	106.9

Supplementary Table 3 (cont.). PA levels during 1st trimester of pregnancy by levels of categorical and ordinal sociodemographic variables.

	SLMPA				OLMPA				SMVPA				OMVPA			
**Civil status**	N	N*	Mean	SD	N	N*	Mean	SD	N	N*	Mean	SD	N	N*	Mean	SD
Married (REF)	184	5	901.9	588.7	189	0	2192.7	567	184	5	348.7	248.9	189	0	259.7	140.9
Living with a partner	82	6	863.3	617.5	88	0	2337.5	571	83	5	346.9	262.5	88	0	**310***	164.9
Divorced or separated	3	1	1400	108.5	4	0	2675	296	3	1	490	434	4	0	364.4	139.7
Single	37	3	748.6	587.6	40	0	2036.1	587.1	37	3	361.1	248.7	40	0	307.7	176.8
Respondent does not answer	4	0	1130	1187	4	0	2535	417	4	0	365	197.6	4	0	208.7	68.1
Unspecified	4	10	468	503	14	0	2628	770	4	10	152.5	147.1	14	0	250	103.2
**Natural pregnancy**																
No (REF)	36	0	889.3	472.5	36	0	2164.6	553.3	36	0	349.3	250.1	36	0	276.8	158.6
Yes	267	14	866.5	611.6	281	0	2231.8	575.4	268	13	348.5	252.9	281	0	280.9	151.9
Unspecified	11	11	1059	807	22	0	2461	760	11	11	352.7	268.5	22	0	252.9	140.7
**Body Mass index**																
Underweight (REF)	10	0	1303	559	10	0	2286	559	10	0	513.5	245.9	10	0	295.7	167.6
Normal	217	9	896.7	611.5	226	0	2214.3	555.2	218	8	368.3	251.1	226	0	286	153
Overweight	52	3	**790.8***	537.5	55	0	2368.4	640.2	52	3	**276.7***	256.6	55	0	281.6	150.7
Obese	20	3	834	705	23	0	2014	557	20	3	320.8	255.7	23	0	231.7	162.1
Unspecified	15	10	642	485	25	0	2372	737	15	10	241.7	159.5	25	0	242.6	121.3
**Number of children**																
One (REF)	165	6	758	506.9	171	0	2033.7	531.6	166	5	368.9	250.1	171	0	279.5	149
Two	118	7	907.1	569.3	125	0	**2371.9***	515.8	118	7	317.5	247.3	125	0	273.9	154.6
Three	24	2	**1475***	953	26	0	**2686***	581	24	2	379.2	264.7	26	0	310.2	175.7
Four or more	5	0	1319	381	5	0	2525	810	5	0	353	388	5	0	257	154.7
Unspecified	2	10	455	544	12	0	2705	807	2	10	140	99	12	0	257.1	110.5

Supplementary Table 3 (cont.). PA levels during 1st trimester of pregnancy by levels of categorical and ordinal sociodemographic variables.

	SLMPA				OLMPA				SMVPA				OMVPA			
**Country of origin**	N	N*	Mean	SD	N	N*	Mean	SD	N	N*	Mean	SD	N	N*	Mean	SD
Spain (REF)	270	7	833	559.7	277	0	2191.9	556.9	270	7	351.7	250	277	0	279.6	152.7
America	32	7	**1240***	834	39	0	**2486***	667	33	6	317.4	248.2	39	0	260.6	156
Other	7	1	791	604	8	0	1994.8	241.9	7	1	294	296	8	0	328.4	161.2
Unspecified	5	10	975	586	15	0	2609	775	5	10	471	372	15	0	281.8	119.8
**Working situation**																
Employed and active (REF)	263	9	810.8	32.3	272	0	2234.8	36.2	264	8	340.7	15.3	272	0	275.8	9.3
Unemployed	20	1	1098	176	21	0	2027.6	99.9	20	1	409.8	63.5	21	0	280.1	32.8
Student	2	1	1820	940	3	0	2118	251	2	1	290	40	3	0	337.9	30.1
Domestic work only	13	2	**1632***	254	15	0	2522	118	13	2	394.6	75.1	15	0	244.1	31.7
Employed on maternal leave	15	2	942	187	17	0	2119	109	15	2	385.7	72.9	17	0	353.5	43.7
Unspecified	1	10	840	*	11	0	2594	224	1	10	210	*	11	0	262	34.5

Note: * = $p < .05$, ** = $p < .01$ and *** = $p < .001$. SLMPA = Self-reported Light to Moderate Physical Activity, SMVPA = Self-reported Moderate to Vigorous Physical Activity, OLMPA = Objective Light to Moderate Physical Activity, OMVPA = Objective Moderate to Vigorous Physical Activity.

Supplementary Table 4. 2nd trimester PA levels and continuous sociodemographic variables.

**Variable**	**N**	**N***	**Mean**	**SE Mean**	**SD**	**Min.**	**Max.**	**Q1**	**Median**	**Q3**
SLMPA	158	195	975.5	49.8	626.2	0	4080	528.8	900	1287.5
OLMPA	166	187	2242.9	50.4	649.3	938	6501.3	1830.4	2167.8	2575.3
SMVPA	158	195	425.9	20.5	258	0	1110	210	390	620
OMVPA	166	187	295.2	13.1	169.1	35	1172	180.5	266	384.4
Age	338	15	33.8	0.2	4.4	21.5	45	30.6	33.8	36.6
Weight prior to pregnancy	317	36	63.3	0.7	12.2	42.4	123	54	61	69
Weight in the second trimester	153	200	67.1	0.9	10.7	46.8	101.6	59	66	73
Height	328	25	1.6	0	0.1	1.4	1.8	1.6	1.6	1.7
Sleep (hours/day)	161	192	7.9	0.1	1.6	0	12	7	8	9
Siesta (hours/day)	148	205	25.3	2.5	29.8	0	150	0	20	45
TV habit (hours/day)	161	192	8.7	0.6	7.4	0	40	3	7	13.5

Note: Mean, standard error, standard deviation, minimum and maximum, Q1, median and Q3 scores for study variables. N = Number of cases, N* = missing cases, SLMPA = Self-reported Light to Moderate Physical Activity, SMVPA = Self-reported Moderate to Vigorous Physical Activity, OLMPA = Objective Light to Moderate Physical Activity, OMVPA = Objective Moderate to Vigorous Physical Activity.

Supplementary Table 5. Spearman correlations between 2nd trimester PA levels and continuous sociodemographic variables.

	**SMVPA**	**SLMPA**	**OMVPA**	**OLMPA**
SLMPA	**.59*** **			
OMVPA	**.46*** **	.09		
OLMPA	.05	**.31*** **	**.19***	
Age	< .01	.13	-.06	**.19***
Weight prior to pregnancy	-.05	-.05	.05	-.08
Weight in the second trimester	-.03	-.05	.08	-.04
Height	.05	-.13	.05	**-.22** **
Sleep	.07	.02	.08	.02
Siesta	-.03	-.12	.07	-.05
TV habit	**.19***	.11	-.03	-.09

Note: Statistically significant values are highlighted in bold. * = p < .05, ** = p < .01 and *** = p < .001. *SLMPA* = Self-reported Light to Moderate Physical Activity, *SMVPA* = Self-reported Moderate to Vigorous Physical Activity, *OLMPA* = Objective Light to Moderate Physical Activity, *OMVPA* = Objective Moderate to Vigorous Physical Activity.

Supplementary Table 6. PA levels during 2nd trimester of pregnancy by levels of categorical and ordinal sociodemographic variables.

	SLMPA				OLMPA				SMVPA				OMVPA			
**Contact with family/friends**	**N**	**N***	**Mean**	**SD**	**N**	**N***	**Mean**	**SD**	**N**	**N***	**Mean**	**SD**	**N**	**N***	**Mean**	**SD**
Daily contact (REF)	75	101	1045.3	694	79	97	2263.7	540.9	75	101	441.7	259.4	79	97	321.7	184.3
Usual contact	57	72	890.4	507.2	59	70	2160.9	466.1	57	72	432	277	59	70	288	137.4
Occasional contact	8	9	905	504	10	7	2276	774	8	9	312.5	156.5	10	7	192.6	105.7
Unfrequent contact	1	3	2640	*	1	3	2457.9	*	1	3	330	*	1	3	58.6	*
Respondent does not answer	2	0	240	42.4	2	0	3197	1781	2	0	240	42.4	2	0	306	49.6
Unspecified	15	10	975	602	15	10	2292	1288	15	10	414.7	238.6	15	10	266.2	213.5
**Preference for exercise**																
Never (REF)	6	26	557	308	7	25	1931.8	191.2	6	26	221.7	121.4	7	25	256.2	85.8
Rarely	32	53	839	666	34	51	2269	724	32	53	292.8	190.3	34	51	237.3	119.5
Sometimes	63	69	1001.7	498.4	67	65	2235.5	544.7	63	69	447.4	255.3	67	65	294.3	164
Often	28	21	942.9	521.3	29	20	2213	402.4	28	21	**495***	222.3	29	20	377.6	206.2
Always	12	16	**1484***	1156	12	16	2378	517	12	16	**632***	380	12	16	332.7	143
Unspecified	17	10	978	563	17	10	2303	1225	17	10	409.4	224.2	17	10	263.1	202.7
**Preference for walking**																
Never (REF)	0	4	*	*	0	4	*	*	0	4	*	*	0	4	*	*
Rarely	12	34	744	634	14	32	1999	664	12	34	274.2	227.9	14	32	196.3	88.3
Sometimes	47	76	921.4	568.8	50	73	2259.6	506	47	76	340.5	226.1	50	73	279.9	120.9
Often	63	46	1005.5	662.9	65	44	2245.6	546.4	63	46	**504***	257.5	65	44	**330.7***	194.7
Always	16	16	1088	727	16	16	2327	642	16	16	470.9	305.8	16	16	321.1	187.2
Unspecified	20	19	1058	558	21	18	2293	1118	20	19	435	227.1	21	18	267.6	186.1

Supplementary Table 6 (cont.). PA levels during 2nd trimester of pregnancy by levels of categorical and ordinal sociodemographic variables.

	SLMPA				OLMPA				SMVPA				OMVPA			
**Alcohol drinking**	**N**	**N***	**Mean**	**SD**	**N**	**N***	**Mean**	**SD**	**N**	**N***	**Mean**	**SD**	**N**	**N***	**Mean**	**SD**
Abstemious (REF)	8	64	1079.4	690	56	63	2268.9	515	55	64	451.5	250.8	56	63	310.4	129.3
Occasional drinker	76	105	948.9	606.3	83	98	2252.3	581.4	76	105	413.3	268.1	83	98	282.9	175.6
Weekly drinker	11	16	630	351	11	16	1999	566	11	16	395.5	283.9	11	16	345.7	237.4
Daily drinker	1	0	1100	*	1	0	1954.4	*	1	0	470	*	1	0	340.2	*
Unspecified	15	10	975	602	15	10	2292	1288	15	10	414.7	238.6	15	10	266.2	213.5
**Tobacco smoking**																
Never Smoker (REF)	82	100	973.2	716	88	94	2224.3	580	82	100	419.7	259.5	88	94	285.1	144.1
Current smoker	5	14	1017	258	5	14	2256	604	5	14	387	258	5	14	213.9	102.8
Non-daily smoker	2	5	612.5	123.7	2	5	2673	587	2	5	298	322	2	5	283.9	12.6
Ex-smoker	54	66	988.9	521.3	56	64	2242.5	519.1	54	66	446.7	266.5	56	64	326.4	196.5
Unspecified	15	10	975	602	15	10	2292	1288	15	10	414.7	238.6	15	10	266.2	213.5
**Car ownership**																
No car (REF)	10	15	1182	744	11	14	2117	452	10	15	401.5	241.5	11	14	303	186.8
One car	60	88	986.9	680	63	85	2281.5	611.4	60	88	449.4	232.4	63	85	306.5	168.2
Two or more cars	73	82	938.2	573.6	77	78	2219.8	522.7	73	82	412.1	286	77	78	290.4	160.3
Unspecified	15	10	975	602	15	10	2292	1288	15	10	414.7	238.6	15	10	266.2	213.5
**Education level**																
No education (REF)	0	2	*	*	0	2	*	*	0	2	*	*	0	2	*	*
Primary education	6	12	1630	1337	6	12	2552	585	6	12	385	293	6	12	288.1	116.9
Secondary education	29	57	**1032**	648	31	55	2397	698	29	57	431.4	296.7	31	55	265.5	111.1
Tertiary education	107	112	**922**	556.1	113	106	2178.8	501.6	107	112	428	252.3	113	106	307.3	178.9

Unspecified	16	12	985	583	16	12	2281	1245	16	12	416.9	230.7	16	12	269.7	206.7

Supplementary Table 6 (cont.). PA levels during 2nd trimester of pregnancy by levels of categorical and ordinal sociodemographic variables.

	**SLMPA**				**OLMPA**				**SMVPA**				**OMVPA**			
**Civil status**	**N**	**N***	**Mean**	**SD**	**N**	**N***	**Mean**	**SD**	**N**	**N***	**Mean**	**SD**	**N**	**N***	**Mean**	**SD**
Married (REF)	89	99	988.7	575.9	93	95	2238.9	542.3	89	99	408.9	254.7	93	95	273.1	138.6
Living with a partner	38	50	978	761	41	47	2290.7	596	38	50	457.2	303.6	41	47	323.9	193.5
Divorced or separated	1	3	525	*	1	3	2258	*	1	3	525	*	1	3	275	*
Single	14	26	908	654	15	25	2095	573	14	26	451.4	187	15	25	381.7	209.1
Respondent does not answer	0	4	*	*	0	4	*	*	0	4	*	*	0	4	*	*
Unspecified	16	13	985	583	16	13	2281	1245	16	13	416.9	230.7	16	13	269.7	206.7
**Natural pregnancy**																
No (REF)	13	23	984	385	14	22	2301	392	13	23	427.7	320.5	14	22	267.9	97.3
Yes	127	153	966.5	656.3	134	146	2212.3	550.9	127	153	428.6	255.1	134	146	300.9	171.3
Unspecified	18	19	1033	566	18	19	2425	1247	18	19	405	243.9	18	19	273.2	198.3
**Body Mass index**																
Underweight (REF)	5	5	1205	523	5	5	2422	696	5	5	497	254	5	5	184.9	95.1
Normal	104	121	967	637.5	109	116	2228.2	550.2	104	121	443.2	261.8	109	116	313.9	172.4
Overweight	26	29	943	662	28	27	2301	616	26	29	366.2	266.6	28	27	275.2	154
Obese	5	18	1020	205.4	5	18	1991	308	5	18	366	239	5	18	256.7	76
Unspecified	18	22	996	649	19	21	2261	1148	18	22	408.9	237.5	19	21	256.1	191.7

Supplementary Table 6 (cont.). PA levels during 2nd trimester of pregnancy by levels of categorical and ordinal sociodemographic variables.

	SLMPA				OLMPA				SMVPA				OMVPA			
**Number of children**	**N**	**N***	**Mean**	**SD**	**N**	**N***	**Mean**	**SD**	**N**	**N***	**Mean**	**SD**	**N**	**N***	**Mean**	**SD**
One (REF)	84	87	894.4	549.7	87	84	2060.1	522.2	84	87	458.3	264	87	84	313.9	160.7
Two	45	79	1060	650.5	49	75	**2479.3***	466.8	45	79	382	241.7	49	75	259	171.4
Three	11	15	1222	1042	12	14	2358	559	11	15	447.7	275.2	12	14	344	164
Four or more	2	3	1050	594	2	3	**3408***	645	2	3	0	0	2	3	277	126.4
Unspecified	16	11	985	583	16	11	2281	1245	16	11	416.9	230.7	16	11	269.7	206.7
**Country of origin**																
Spain (REF)	127	150	944.6	552.5	135	142	2223.7	527.9	127	150	431.3	264.9	135	142	300.6	170.2
America	10	29	**1436***	1283	10	29	2532	888	10	29	373.5	274.2	10	29	251	104.4
Other	4	3	700	437	4	3	2009	493	4	3	370	134.7	4	3	322.2	141.9
Unspecified	17	13	1001	568	17	13	2280	1206	17	13	428.8	228.8	17	13	271.8	200.3
**Working situation**																
Employed and active (REF)	123	149	917.7	564.6	131	141	2236.1	576.1	123	149	406.7	249.1	131	141	297.7	169
Unemployed	7	12	985	530	7	12	2008	311	7	12	518	344	7	12	248.2	49.5
Student	0	3	*	*	0	3	*	*	0	3	*	*	0	3	*	*
Domestic work only	4	11	**2544***	1133	4	11	2739	334	4	11	609	264	4	11	203.8	165.4
Employed on maternal leave	8	10	1054	498	8	10	2235	349	8	10	566	332	8	10	390.5	139.1
Unspecified	16	10	985	583	16	10	2281	1245	16	10	416.9	230.7	16	10	269.7	206.7

Note: * = $p < .05$, ** = $p < .01$ and *** = $p < .001$. SLMPA = Self-reported Light to Moderate Physical Activity, SMVPA = Self-reported Moderate to Vigorous Physical Activity, OLMPA = Objective Light to Moderate Physical Activity, OMVPA = Objective Moderate to Vigorous Physical Activity.
